# Brown fat does not cause cachexia in cancer patients: A large retrospective longitudinal FDG-PET/CT cohort study

**DOI:** 10.1371/journal.pone.0239990

**Published:** 2020-10-08

**Authors:** Anton S. Becker, Caroline Zellweger, Sara Bacanovic, Sabine Franckenberg, Hannes W. Nagel, Lukas Frick, Khoschy Schawkat, Matthias Eberhard, Christian Blüthgen, Jörk Volbracht, Rudolf Moos, Christian Wolfrum, Irene A. Burger

**Affiliations:** 1 Department of Health Sciences and Technology; Institute of Food, Nutrition and Health, ETH Zurich, Schwerzenbach, Switzerland; 2 Institute of Diagnostic and Interventional Radiology, University Hospital Zurich, Zurich, Switzerland; 3 Department of Nuclear Medicine, University Hospital Zurich, Zurich, Switzerland; 4 Department of Pathology and Molecular Pathology, University Hospital Zurich, Zurich, Switzerland; 5 Division of Controlling and Data Management, University Hospital Zurich, Zurich, Switzerland; Medical University of Vienna, AUSTRIA

## Abstract

**Background:**

Brown adipose tissue (BAT) is a specialized form of adipose tissue, able to increase energy expenditure by heat generation in response to various stimuli. Recently, its pathological activation has been implicated in the pathogenesis of cancer cachexia. To establish a causal relationship, we retrospectively investigated the longitudinal changes in BAT and cancer in a large FDG-PET/CT cohort.

**Methods:**

We retrospectively analyzed 13 461 FDG-PET/CT examinations of n = 8 409 patients at our institution from the winter months of 2007–2015. We graded the activation strength of BAT based on the anatomical location of the most caudally activated BAT depot into three tiers, and the stage of the cancer into five general grades. We validated the cancer grading by an interreader analysis and correlation with histopathological stage. Ambient temperature data (seven-day average before the examination) was obtained from a meteorological station close to the hospital. Changes of BAT, cancer, body mass index (BMI) and temperature between the different examinations were examined with Spearman’s test and a mixed linear model for correlation, and with a causal inference algorithm for causality.

**Results:**

We found n = 283 patients with at least two examinations and active BAT in at least one of them. There was no significant interaction between the changes in BAT activation, cancer burden or BMI. Temperature changes exhibited a strong negative correlation with BAT activity (ϱ = -0.57, p<0.00001). These results were confirmed with the mixed linear model. Causal inference revealed a link of Temperature ➜ BAT in all subjects and also of BMI ➜ BAT in subjects who had lost weight and increased cancer burden, but no role of cancer and no causal links of BAT ➜ BMI.

**Conclusions:**

Our data did not confirm the hypothesis that BAT plays a major role in cancer-mediated weight loss. Temperature changes are the main driver of incidental BAT activity on FDG-PET scans.

## Introduction

There are two main types of adipose tissues found in mammals both with distinct localization, physiology and function: white adipose tissue (WAT) and brown adipose tissue (BAT) (summarizing true brown and brite “brown-in-white” adipocytes). White adipocytes are characterized by large lipid-filled vacuoles that store triglycerides and can mobilize these stores through lipolysis to provide energy to the organism [1]. In contrast to white adipocytes, brown adipocytes are enriched in mitochondria, contain multilocular lipid droplets, and regulate whole body thermogenesis by dissipating heat through mitochondrial uncoupling protein 1 (Ucp1) [1–3].

In addition to the thermogenic effects, brown adipocytes have a large capacity for lipid and glucose metabolism as evidenced by their ability to normalize hyperlipidemia and hyperglycemia in mouse models of dyslipidemia and diabetes [4,5]. Consequently, activation of brown and brite adipocytes has been proposed as a strategy to combat obesity-related metabolic dysfunctions [6,7].

Cachexia is characterized by adipose tissue atrophy and skeletal muscle wasting which is observed in many chronic diseases such as sepsis, chronic kidney disease, AIDS, congestive heart failure and cancer [8]. Cachexia is a devastating condition that worsens the outcome of cancer patients and often precludes them from additional therapies; it is estimated that 20% of cancer deaths result from cachexia [9]. Weight loss, adipose tissue and muscle atrophy as well as anorexia are the hallmark symptoms of cancer cachexia. Affected patients are in a state of negative energy balance which cannot be attributed to decreased food intake alone, however. the underlying mechanisms are poorly understood [8]. The loss of fat mass in cachectic patients has been attributed to increased adipocyte lipolysis and increased sensitivity of adipocytes to inflammatory cytokines and lipolytic stimuli [10,11]. Recent studies have suggested that adipose tissue browning leads to higher basal energy expenditure through induction of UCP1 expression in WAT as a potential mechanism promoting cachexia [12,13]. Petruzelli *et al*. demonstrated that browning of subcutaneous WAT is consistently found across various xenograft and genetic models of cancer cachexia in mice [12]. Furthermore, Kir *et al*. and Petruzzelli *et al*. recently identified parathyroid hormone related protein (PTHrP) and interleukin-6 (IL-6), respectively [12,13] as two tumor-derived factors that influence adipose tissue browning in cancer cachexia. They demonstrated these factors have direct effects by inducing thermogenic genes in primary adipocytes and promoting development of brite cells within WAT of mouse models of cancer cachexia. These studies have gained widespread acceptance [14], however they warrant further investigation as 1) the cellular origin of the brite cell in cachexia is not yet established 2) neutralization of either PTHrP or IL-6 is not sufficient to inhibit cachexia in tumor-bearing mice [12,13] 3) recent experiments by Rohm et al. suggest that other mediators in the white adipose tissue are the dominant factors contributing to cachexia independent of adipose tissue browning [15]. Here we present a retrospective longitudinal cohort study investigating associations between the evolution of cancer, BAT activation and changes in BMI.

## Methods

This retrospective study was approved by the local ethics committee (KEK-ZH # 2015–0282), who waived the need for informed consent.

### Study cohorts and data sources

For a first pilot study, we examined all patients undergoing FDG-PET/CT at our institution between November 2014 and February 2015 (Cohort I). After examining the preliminary results of this pilot cohort, we sought to validate our findings in an extended retrospective study, encompassing all FDG-PET/CT examinations from 2007–2015 (Cohort II) in the winter months (November-February). We selected the winter months to optimize the manual readout, since BAT has a propensity to be more easily activated during the cold season [16]. For patients with active BAT, all available examinations in any month (i.e. outside the Nov-Feb-window) were included. All data was extracted from the radiology information system of the hospital and directly from the metadata of the DICOM images. Patient height and weight at the time of the examination was measured for each examination and saved in the DICOM image metadata. The indication for the examination was extracted from the radiology report, coded in ICD-10 format. For a subset of patients, histopathological cancer staging data was collected from a certified (according to the guidelines of the German cancer society DKG) cancer registry “Comprehensive Cancer Center Zurich”. To account for ambient temperature differences between the examinations, the daily average temperature data 2005–2016 was obtained from a weather station near the hospital from the Swiss Federal Office of Meteorology and Climatology MeteoSwiss.

### BAT and cancer quantification

**Fig 1:**
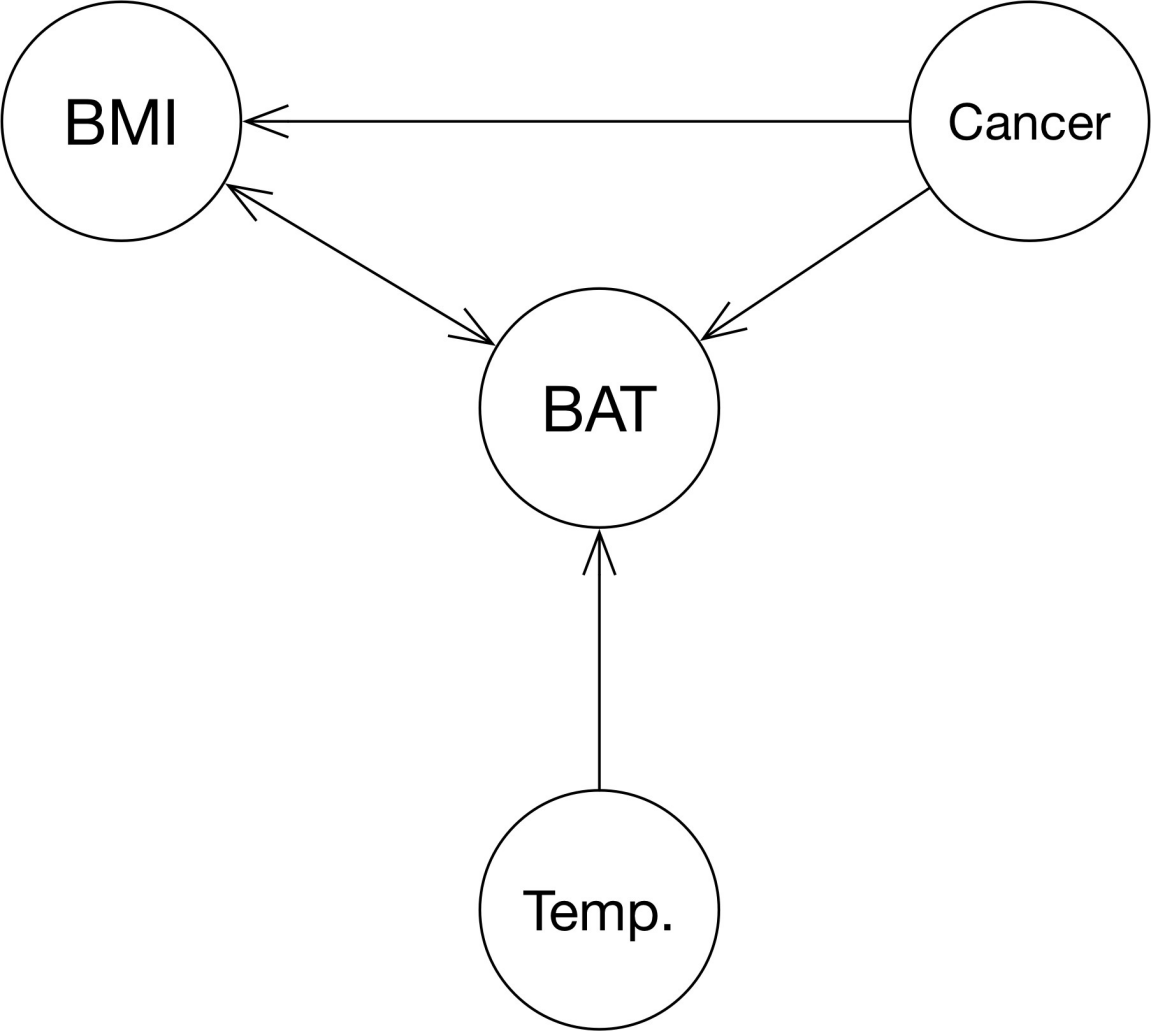
Directed acyclic graph (DAG) representation of the hypothesis that BAT is a mediator of cachexia in cancer patients, including ambient temperature (Temp.) as a potential known confounder. Age and sex have been omitted in this graph for simplicity since they are known and, more importantly, non-modifiable factors.

For quantification of BAT and cancer activity, three radiologists examined the images on a standard clinical radiology workstation. BAT activity was graded into four categories (0–3) as described before [17,18]. In short, BAT depots are divided into three anatomical zones: cervical-supraclavicular, thoracic and abdominal. BAT in humans is activated in a cranio-caudal sequence, i.e. the most caudally located active depot will be indicative of the total strength of BAT activation (Fig 1a).

In all patients exhibiting BAT activity, the total burden of metabolically active cancer was also assessed by a five-tier grading system as follows (Fig 1b): 0 = No visible signs of cancer, 1 = cancer visible in CT, but metabolically inactive in FDG-PET, 2 = single lesion, 3 = local metastases, 4 = distant metastases. Category 2 single lesions were upgraded if highly metabolically active (defined as a [maximum standardized uptake value] SUV_max_ > 15 mg/dl). In patients with multiple examinations, all available/subsequent examinations were considered when assessing whether the metabolic activity truly represented active cancer or not.

This grading system for cancer burden estimation was validated two-fold: First, four radiologists evaluated 500 cases and we assessed the interreader agreement with absolute agreement, the concordance correlation coefficient (CCC) and pair-wise weighted Cohen’s kappa (κ). CCC and κ-Scores were interpreted as follows: slight (< 0.20), fair (0.20–0.39), moderate (0.40–0.59), substantial (0.60–0.79), and excellent (> 0.80) agreement. Second, we correlated the gradings with histopathological data from the cancer registry.

For estimation of the cancer burden in the cohort without active BAT (BAT-negative), we applied a state-of-the-art natural language processing pipeline with machine learning on the radiological reports implemented in Python version 3.6 using the natural language toolkit version 3.2.5. Words were stemmed with a German word stemming function (removing endings such as plural and declinations) and vectorized. We empirically tested various machine learning classifiers from the package Scikit-learn version 0.19.1 (Naïve Bayes, Adaboost, K-Nearest Neighbors, Random Forest, Support vector machine (SVM)). We found the SVM with a linear kernel worked best, and hence trained an SVM on the manually read cases (n = 1738). 5-fold cross-validation with 20% of the training data withheld from the classifier in each run showed an area under the curve of 0.71 (averaged over all five classes), which was satisfactory for our purposes.

### Cross-sectional analysis

We compared all underweight patients in the BAT-positive and BAT-negative groups with a chi-square test. Various definitions for cachexia exist in the literature; in a recent consensus paper, Fearon & Strasser et al. propose a combination of BMI < 20 kg/m^2^ and weight loss >2% over six months [19]. We opted to use a slightly more stringent threshold of BMI < 18.5 kg/m^2^ under omission of weight loss for our cachexia analysis, since weight loss data was only available for the minority of subjects. Next, we examined the sex and age differences between the two groups, since it is known that females and younger individuals are more likely to exhibit BAT activation [6]. In the pilot study, we qualitatively compared the 25 most prevalent cancer types as coded by ICD-10 between the groups. Conspicuous differences were examined with a chi-square test. Furthermore, in the extended study, we examined the influence of different cancer types, age and sex on BAT activation with a multivariate analysis (general linear model).

### Longitudinal and subgroup analysis

Since the number of and interval between examinations was heterogeneous among patients in this retrospective cohort, we standardized the data by determining the median overall follow-up interval and selecting the two examinations with the interval closest to the median. We used the daily temperature to account for short-term effects as well as the mean over the last week (seven days) before the actual examination in order to account for the long-term effect of atmospheric temperature changes on BAT as proposed by Senn et al. [20]. We ignored the ambient temperature since patients are kept at thermoneutral conditions prior to the FDG-PET/CT examination to avoid BAT activation, which can interfere with diagnostic accuracy of the PET component. The differences between the second and first examination of BAT, cancer grade, BMI and temperature were assessed with Spearman correlation testing (Spearman’s rho = ϱ). We generated 10 000 samples using bootstrapping to obtain confidence intervals for the correlation coefficients. Next, we examined the whole longitudinal data set with a mixed linear model, once with BAT only, and once with BAT*BMI as a target.

Lastly, we performed causal inference analysis (package *pcalg* v. 2.6–10) using the Peter and Clark-Algorithm (PC-Alg.) to examine any causal effects that can be deducted from the data. If the hypothesis was to be confirmed, the resulting graph would have to resemble Fig 1.

Despite the known risk of finding false positives when performing subgroup analyses, we chose three subgroups based on known clinical parameters that could help to find a potential association between cancer and BAT activity. In the first subgroup analysis, we analyzed all patients who had lost weight during a specific interval. Second, we analyzed different groups of cancers which are known to cause cachexia (ICD-10 code given in brackets): Lung cancer (C34), melanoma (C43), esophageal and stomach cancer (C15-16), pancreatic (C25) as well as head and neck cancer (C01, 04, 09, 10).

All analyses were performed in R version 4.0.0. The full raw data in csv-format and statistical analysis are available in an online repository on https://github.com/ASBecker/BATcancer. Please note that some noise has been added to the temperature data in order to ensure an adequate level of anonymity: Since the weather station is known, it would be trivial to reverse engineer the examination dates, which together with the other biometric parameters and cancer type would allow for identification of individuals. This may slightly change some decimals of the results, but does not alter the conclusions.

## Results

### Evaluation of cancer grading system

**Fig 2:**
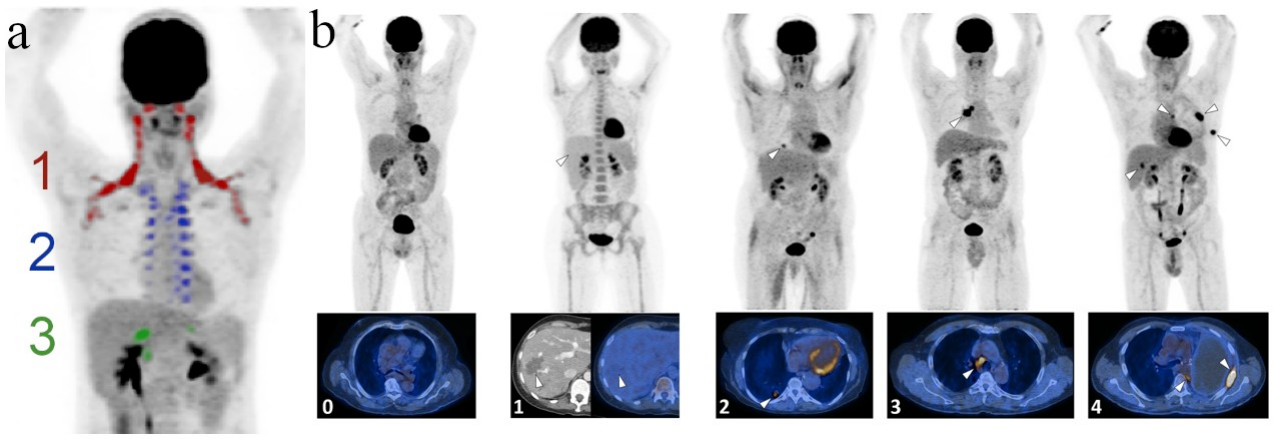
2a: Coronal FDG-PET projection showing the anatomical locations of the BAT depots (color coded: 1 = supraclavicular/cervical, 2 = thoracic, 3 = infradiaphragmatic/abdominal activation). BAT in humans is strictly activated in a craniocaudal fashion, i.e. the more caudally activated depots indicate higher overall glycolysis and can hence be categorically graded in this way. **2b**: **Representative examples of patients suffering from different grades of cancer burden**. Grades were assigned as follows: 0 = No visible signs of cancer, 1 = cancer visible in CT, but metabolically inactive in FDG-PET, 2 = single lesion, 3 = local metastases, 4 = distant metastases. Top row: Maximum intensity projections of the FDG-PET data (dark = high FDG uptake), bottom row: fused PET/CT slices through representative locations (yellow = high FDG-uptake). Index lesions are marked with white arrow tips.

**Fig 3:**
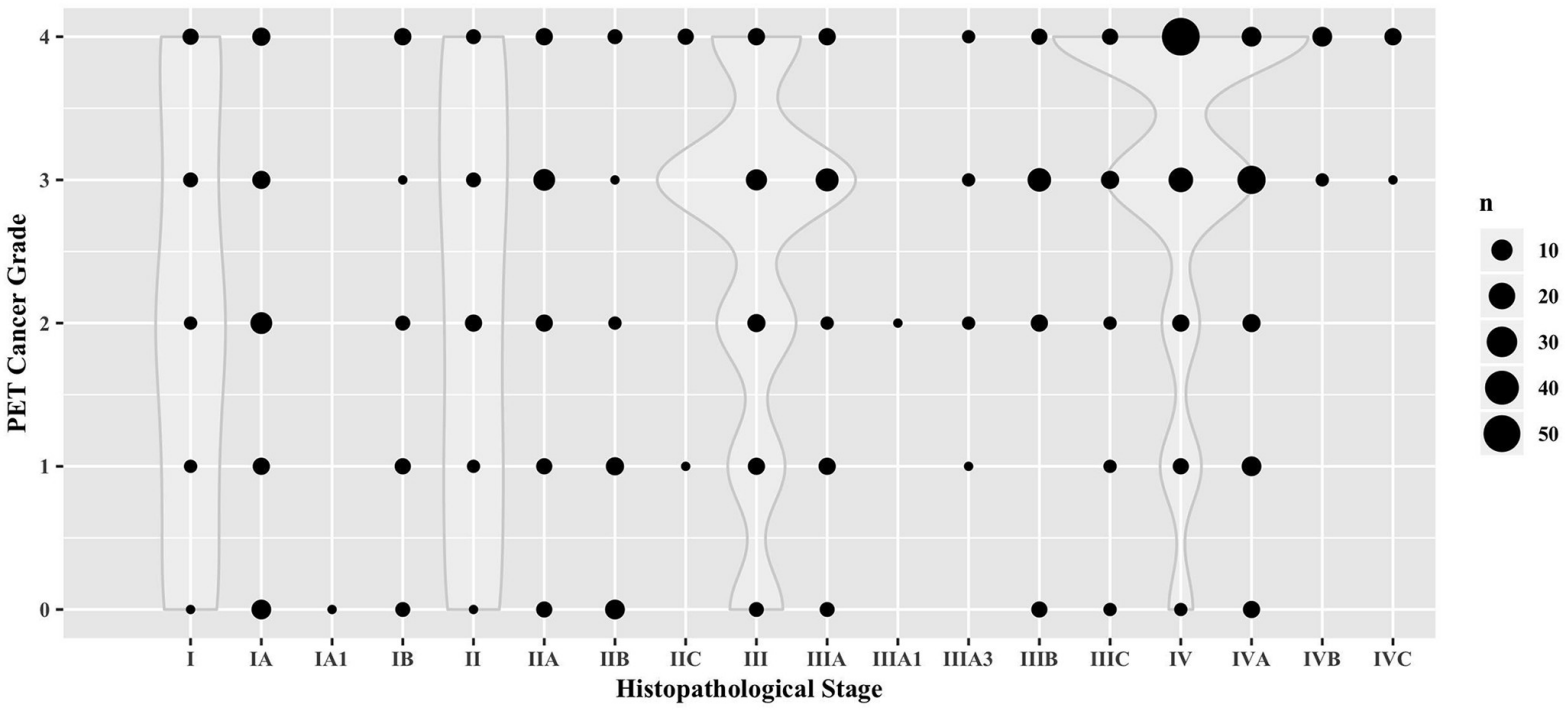
Dotplot depicting the correlation of the cancer stage, manually assigned from the FDG-PET examination report, with the histopathological cancer stage (n = 388; ϱ = 0.30, p<0.00001). Overlaid violin plots depict the density estimation for the main categories I, II, III and IV.

To efficiently grade and compare the total cancer burden in a large, heterogeneous cohort comprising different cancer types, we developed a five-tier semi-quantitative grading system (Fig 2b) to allow for a direct comparability. To validate this system, we first tested the robustness for the subjective assignment to a category with an interreader analysis of radiologists, which showed that in 43.2% of cases, all readers assigned the exact same score. Pairwise absolute agreement ranged from 55.4% to 80.6%. In 84.8% of cases, all four readers agreed within ±1 category. Interreader agreement was excellent for all readers (CCC = 0.86, 95%-CI: 0.83–0.89). Pairwise interreader agreement ranged from substantial (κ = 0.69; 0.56–0.81) to excellent (κ = 0.84; 0.70–0.97). As a second validation step, we correlated the assigned cancer grade with the clinical/histopathological stage in 441 patients receiving an initial staging FDG-PET/CT for which corresponding histopathological staging data was available. Our FDG-PET cancer grading correlated significantly with the grade assigned from histopathological staging (ϱ = 0.22, p<0.00001). This correlation also held true when only analyzing the manually assigned cases (n = 388; ϱ = 0.30, p<0.00001) as shown in Fig 3. These results indicate that it is a suitable approximation for total tumor burden across different cancer types.

### Cohort I

**Fig 4:**
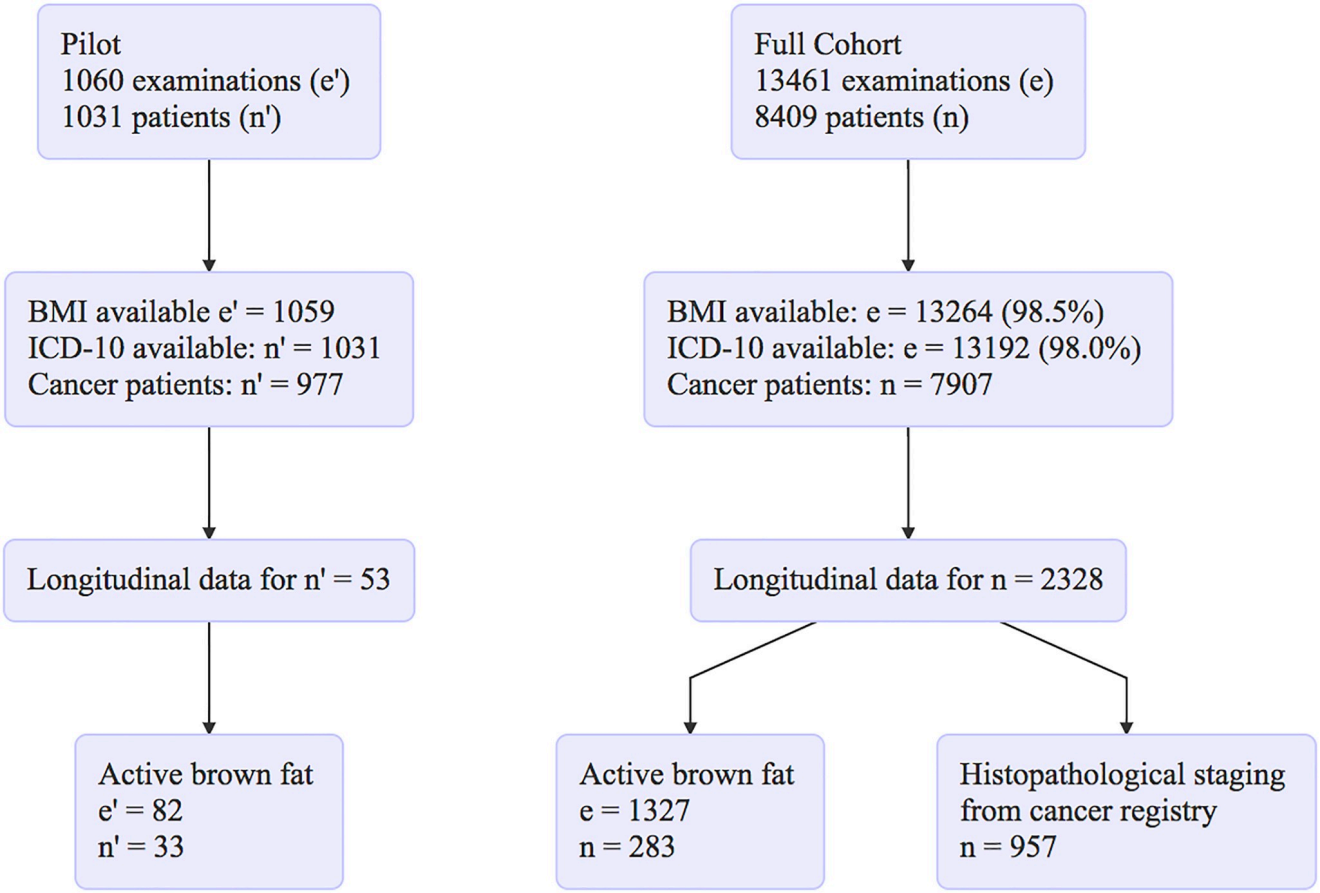
Flowchart illustrating the patient selection process.

**Fig 5:**
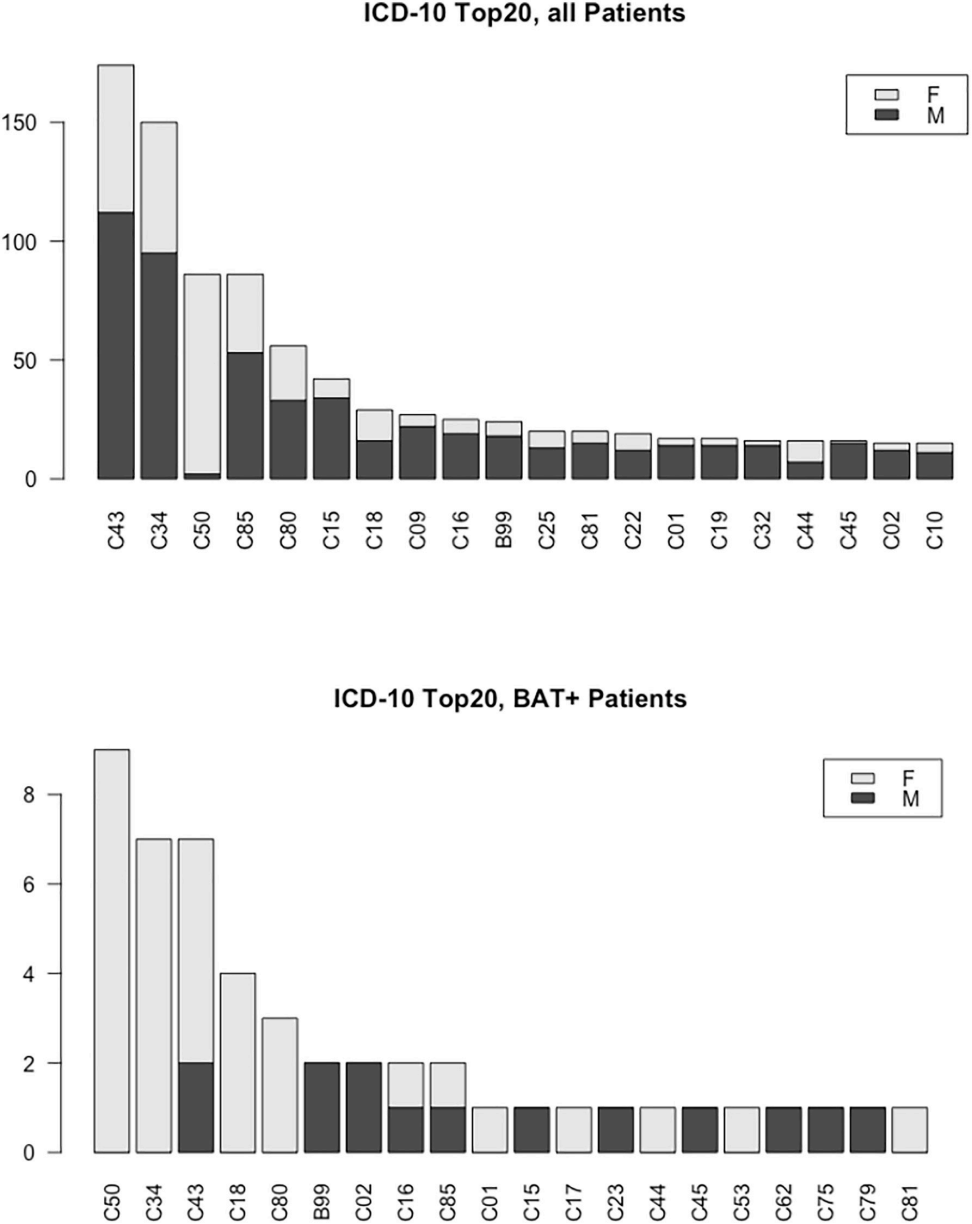
Cancer distribution grouped by ICD-10 in the cohort I (pilot cohort) as well as only in patients with active BAT (BAT+).

To investigate the relationship between cancer burden and BAT activation we performed a pilot study in which we included 1 060 FDG-PET/CT examinations of 1 031 patients (419 females) from Nov 2014 to Feb 2015. Active BAT was found in 53 patients (37 females) (c.f. flowchart in Fig 4). BAT-positive patients were significantly younger than BAT-negative ones (49.32 ± 15.54 vs. 60.85 ± 14.25 years). Based on previous reports, which suggest that BAT contributes to cancer cachexia we next analyzed whether BMI and BAT were interdependent in the patients. Therefore, we analyzed the data in a cross-sectional manner and found significantly fewer patients classified as cachectic (BMI < 18.5) in the BAT-negative vs. the BAT-positive group (7.1% vs. 18.9%, p = 0.004). Since previous reports indicate that the browning of adipose tissue might be dependent on the cancer type, we compared the prevalence of different cancer types between the two groups. We found a higher prevalence of breast cancer (7.1% vs. 18.9%, p = 0.004) and testicular cancer (7.1% vs. 18.9%, p = 0.004) in BAT-positive patients. Since BAT is predominantly found in females and younger patients it is possible that this association might simply be explained by the higher prevalence of females and younger age, in these two cancer groups. In addition, there were also significantly more colon cancers (7.1% vs. 18.9%, p = 0.02) in the BAT-positive group. These results of the pilot study are summarized in Fig 5 and in the flowchart in Fig 4, respectively. In summary, our data of this small pilot with a limited number of cases suggested that BAT is associated with certain cancer types.

**Fig 6:**
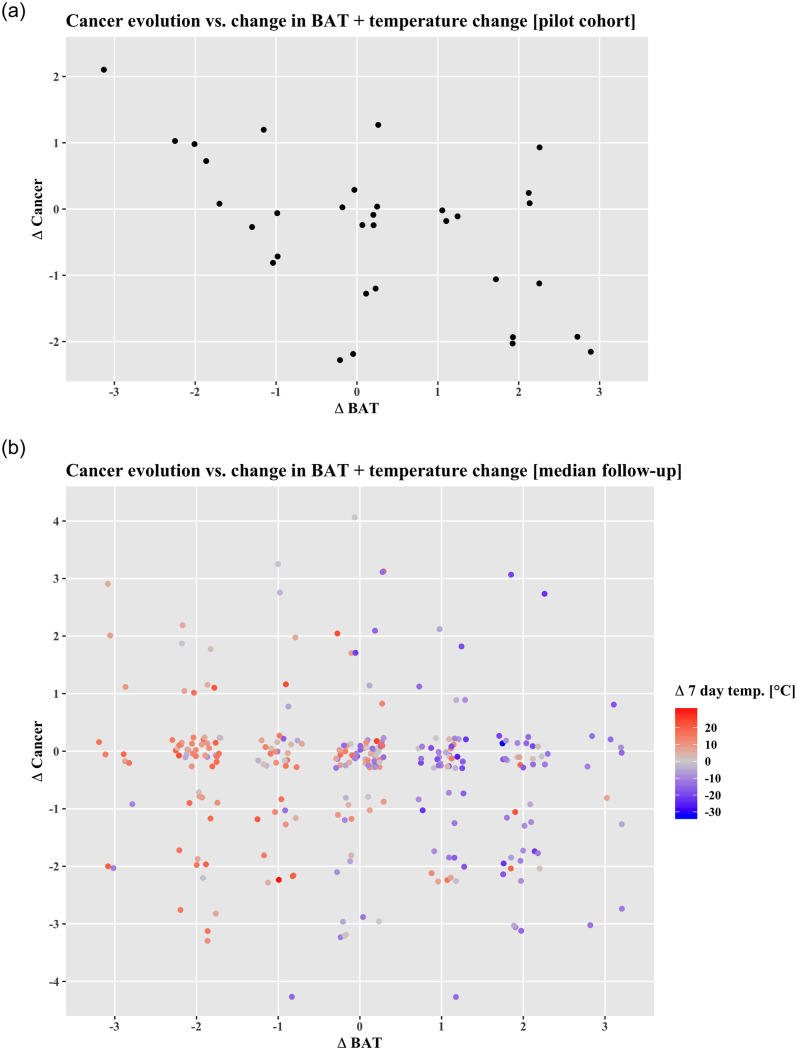
**6a**: Scatterplot showing the (spurious) negative correlation between change in cancer and BAT found in Cohort I (n = 33, ϱ = -0.48, p = 0.005). **6b**: Scatterplot depicting the change in cancer burden (ΔCancer) vs. the change in brown adipose tissue activation (ΔBAT) in Cohort II. No significant correlation was found (n = 280, ϱ = -0.08, p = 0.15). However, when paying attention to the change in daily temperature (average over the 7 days before the respective examination), one can appreciate that BAT activation was strongly dependent on changes in ambient temperature (ϱ = -0.50, p = <0.0001).

Since a cross-sectional analysis is insufficient to propose a cause-effect relationship, we identified patients with multiple scans to obtain data on changes in BAT over time in relation to cancer development. We found previous FDG-PET/CT scans in 33 of the 53 BAT-positive patients, with a median of 142 days between the examinations. To quantify BAT development, we subtracted the BAT and cancer grade as well as BMI in the second examination from the one in the first one and we observed that the change in cancer burden (Δcancer) correlated negatively with the ΔBAT activation as depicted in Fig 6a (ϱ = -0.48, p = 0.005), while no significant correlation between ΔBAT and ΔBMI was found (ϱ = -0.01, p = 0.93). Based on this data from 1031 patients we concluded that with increasing cancer burden, BAT activation is reduced rather than increased.

### Cohort II

To substantiate our findings, we extended our study to include 13 461 examinations of 8 409 patients (3 591 or 42.7% females), examined between November 2007—February 2015. Of those, 7 907 (3 581 or 45.3% females) received an examination due to cancer. We found active BAT in 460 patients (5.8%), of which 330 were females (71.7%). As observed in Cohort I, we found that the BAT positive patients were younger and had a lower BMI than the BAT negative patients (Table 1). While we still found a tendency for more breast and testicular cancer in BAT-positive patients, we could not replicate the previously significant difference in colon cancer prevalence. In addition, we did not find any other conspicuous changes as shown in Table 2. Multivariate analysis revealed only two significant differences between the groups: C47 (malignant neoplasm of the peripheral nerves of head, face and neck) with p = 0.004 and C48 (neoplasm of the retroperitoneum) with p = 0.048, however, given the low number of these pathologies (three and five, respectively), these results should be interpreted with caution. Both age and BMI remained highly associated with BAT activation (p<0.0001), whereas temperature did not. This is not surprising, since we specifically selected most examinations from the winter months, where similar baseline atmospheric temperatures are prevalent.

**Table 1:** Demographic characteristics of the study cohort (patients receiving FDG-PET/CT due to cancer). Data are mean (SD) or counts (%). P-values are derived from a Welch two-sample t-test and chi-square test, respectively.

	ALL	BAT-NEGATIVE	BAT-POSITIVE	*P*-VALUE
N	7907	7447 (94.2%)	460 (5.8%)	-
AGE [Y]	59.6 (14.5)	60.4 (14.0)	46.4 (16.4)	<0.0001
FEMALES	3581 (45.3%)	3251 (43.7%)	330 (71.7%)	<0.0001
BMI [KG/M^2^]	23.0 (5.3)	23.1 (5.4)	21.1 (4.6)	<0.0001

BAT = Brown Adipose Tissue; BMI = Body Mass Index.

**Table 2:** Cancer distribution in the whole cohort grouped by ICD-10 in the patients with (positive) and without (negative) active BAT. The 25 most frequently occurring cancers in BAT-positive patients are shown. Data are presented as counts and percentages. Percentages are referring to the respective subgroup (BAT-positive or negative).

ICD-10		BAT-NEG.		BAT-POS.	
N		7447	*100%*	460	*100%*
C50	Breast	736	*9*.*9%*	73	*15*.*9%*
C34	Lung	1358	*18*.*2%*	61	*13*.*3%*
C43	Melanoma	808	*10*.*9%*	48	*10*.*4%*
C81	Hodgkin	334	*4*.*5%*	45	*9*.*8%*
C18	Colon	422	*5*.*7%*	21	*4*.*6%*
C85	Non-Hodgkin Lymphoma	268	*3*.*6%*	20	*4*.*3%*
C80	Unknown Primary	369	*5%*	19	*4*.*1%*
C62	Testicular	88	*1*.*2%*	18	*3*.*9%*
C82	Follicular Lymphoma	301	*4%*	16	*3*.*5%*
C53	Cervix Uteri	63	*0*.*8%*	15	*3*.*3%*
C49	Soft tissue	64	*0*.*9%*	9	*2%*
C73	Thyroid	74	*1%*	8	*1*.*7%*
C01	Tongue base	160	*2*.*1%*	7	*1*.*5%*
C16	Stomach	140	*1*.*9%*	7	*1*.*5%*
C09	Tonsils	113	*1*.*5%*	6	*1*.*3%*
C15	Esophagus	280	*3*.*8%*	6	*1*.*3%*
C20	Rectum	93	*1*.*2%*	6	*1*.*3%*
C25	Pancreas	161	*2*.*2%*	6	*1*.*3%*
C41	Bone/Cartilage	24	*0*.*3%*	6	*1*.*3%*
C21	Anus	41	*0*.*6%*	4	*0*.*9%*
C22	Liver	147	*2%*	4	*0*.*9%*
C45	Mesothelioma	93	*1*.*2%*	4	*0*.*9%*
C56	Ovary	78	*1%*	4	*0*.*9%*
C10	Mesopharynx	114	*1*.*5%*	3	*0*.*7%*
C02	Tongue	54	*0*.*7%*	2	*0*.*4%*

To further investigate the cause-effect relationship between BAT and cancer, we performed a longitudinal analysis analogous to the one in Cohort I. Multiple examinations were available for 2328 cancer patients, the median was two examinations per patient (IQR = 2–4, 95%-CI: 2–8 exams). Of these, 283 patients had active BAT in at least one examination, with a median follow-up interval of 195 days. We could not validate the previously observed significant negative correlation between cancer burden and BAT activation change (ϱ = -0.10, p = 0.08), albeit the direction of the effect remained. Bootstrapped 95%-CI for ϱ values were -0.22–0.02 for Δcancer—ΔBAT, -0.10–0.14 for ΔBAT–ΔBMI and -0.13–0.12 for Δcancer–ΔBMI. Only the Δambient temperature correlated significantly with ΔBAT (both daily and 7-day mean: ϱ = -0.58, p = <0.00001). The first mixed linear model showed a significant correlation between BAT and BMI (p = 0.04), hence, for the second model, BAT*BMI was chosen as a target. In neither of the analyses was cancer found to have a significant influence on BAT (and/or BMI), however, the known factors age, sex and temperature did exhibit a significant influence as summarized in Fig 6b and Tables 3 and 4.

**Table 3:** Mixed linear model analysis with the formula BAT~Cancer+BMI+Sex+Age+Temperature+(1|Patient-ID) showing no influence of cancer on BAT activation grade.

	BAT		
	*B*	*CI*	*p*
**Fixed Parts**			
(Intercept)	1.97	1.71–2.24	< .001
Cancer	-0.02	-0.06–0.01	0.173
BMI	-0.01	-0.02–0.00	0.084
Sex (M)	-0.32	-0.43 –-0.20	< .001
Age	-0.01	-0.02 –-0.01	< .001
Temperature	-0.04	-0.05 –-0.04	< .001
**Random Parts**			
σ^2^	0.749		
τ_00,Pat.ID_	0.125		
N_Pat.ID_	661		
ICC_Pat.ID_	0.143		
Observations	1769		
R^2^/Ω_0_^2^	0.351/0.319		

**Table 4:** Mixed linear model analysis with the formula BAT*BMI~Cancer+Sex+Age+Temperature+(1|Patient-ID) showing no influence of cancer on BAT/BMI.

	BAT * BMI		
	*B*	*CI*	*p*
**Fixed Parts**			
(Intercept)	36.37	32.21–40.53	< .001
Cancer	-0.43	-1.18–0.32	0.260
Sex (M)	-6.63	-9.16 –-4.11	< .001
Age	-0.23	-0.30 –-0.16	< .001
Temperature	-0.91	-1.04 –-0.79	< .001
**Random Parts**			
σ^2^	335.647		
τ_00,Pat.ID_	77.062		
N_Pat.ID_	661		
ICC_Pat.ID_	0.187		
Observations	1769		
R^2^/Ω_0_^2^	0.396/0.352		

Since most reports demonstrated an association between cachexia and BAT, we performed a subgroup analysis to assess whether patients who had lost weight, or suffered from certain types of known cachectogenic cancers, showed higher BAT activation. Neither the subgroup analysis of patients who had lost weight during the follow-up interval (n = 126), nor the analysis of the different groups of cancers revealed any significant correlation, as summarized in Table 5.

**Table 5:** Spearman correlation coefficients and corresponding p-values for two different subgroups (raw p-values, not corrected for multiple comparisons). The first subgroup is comprised of all patients who had lost weight. The second subgroup are selected cancers known to cause cachexia (ICD-10: C43, C34, C25, C16, C15, C01, C04, C09, C10).

	RHO	P-VALUE
**SUBGROUP: WEIGHT-LOSS**		
**ΔBAT-ΔCANCER**	-0.10	0.08
**ΔCANCER-ΔBMI**	-0.01	0.88
**ΔBAT-ΔBMI**	0.02	0.88
**ΔBAT-ΔTEMPERATURE**	-0.57	<0.00001
**SUBGROUP: SELECTED CANCER TYPES**		
**ΔBAT-ΔCANCER**	0.02	0.84
**ΔCANCER-ΔBMI**	-0.03	0.78
**ΔBAT-ΔBMI**	-0.13	0.78
**ΔBAT-ΔTEMPERATURE**	-0.56	<0.00001

**Fig 7:**
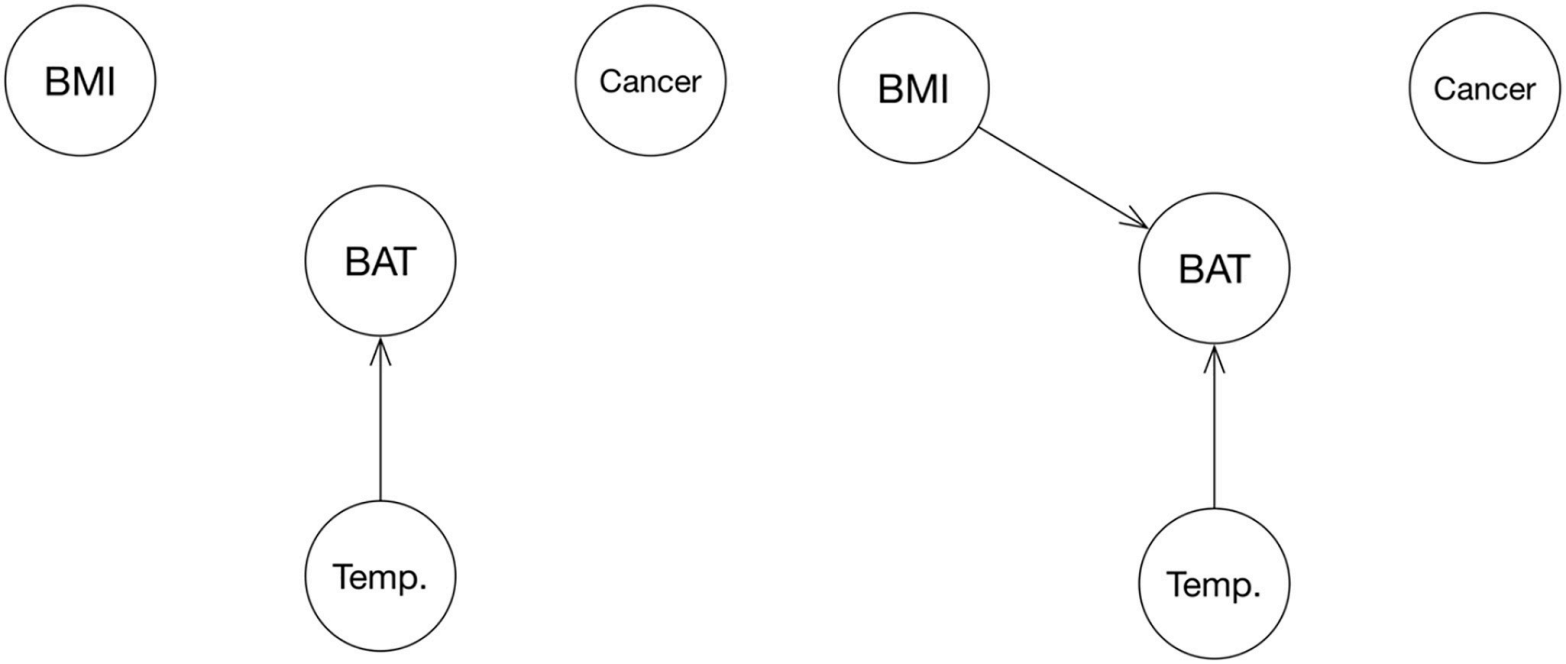
DAG derived from full cohort (left) and from subgroup who exhibited weight loss (right), showing no causal link between cancer, BAT and weight loss. The arrow from BMI to BAT implicates that if there is a causal link between the two, it is lower BMI that causes BAT to become more active and not the other way around.

Causal inference analysis yielded a link between Temperature and BAT change when considering the full cohort, and a link from BMI and Temperature change to BAT when considering only patients who lost weight (Fig 7).

Overall, our analysis suggests that the baseline age, sex and BMI as well as changes in ambient temperature, possibly also in BMI, are the main variables influencing BAT activation. There was no indication for a causal link involving BAT-mediated cancer cachexia.

## Discussion

The first observations that BAT activity might be associated with the hypermetabolic state and weight loss in cancer patients was published in 1981 [21]. This was confirmed by Shellock et al, who detected BAT in 80% of cancer patients versus only in 13% in age matched non-cancer patients [22]. With the introduction of combined FDG-PET with CT, the frequently observed high FDG activity in the supraclavicular area could be co-registered to fatty tissue and identified as active BAT [23]. These observations led to several retrospective studies investigating the presence and distribution of BAT activity in large cohorts, identifying strong associations between BAT activity with sex, age, BMI and fasting glucose levels [6,24–26]. Furthermore, some retrospective cross-sectional studies suggested a correlation between the volume of active BAT and cancer [27–31], while others did not find this association [6,16]. Of note, none of those studies accounted for outside temperature, which may have been rooted in the assumption that humans live exclusively at thermoneutrality. The strong statistical signal of ambient temperature causing BAT activation in our cohort, on the other hand, favors the notion that humans are “skirting” at the border between thermoneutrality and mild cold exposure.

Our retrospective analysis of 13 461 PET scans in patients with various levels of tumor burden confirmed the previously noted association of low BMI and high BAT activity. However, the intra-individual, longitudinal analysis of multiple PET scans in 283 patients did not show any correlation between changes in cancer burden and BAT activity or BMI. These results stand in contrast with the hypothesis that tumor induced cachexia is mediated by increased activation of BAT activity [22,30]. The large cohort allowed us to analyze cancer subtypes known to cause tumor induced cachexia, such as pancreatic, lung or upper gastrointestinal cancer compared to tumors less frequently associated with cachexia (e.g. breast and lower intestinal cancer) [9,32]. However, no association between cancer subtypes and increased BAT activity was found.

These results contradict previous observations that suggested an association between BAT activation and cancer cachexia [32], but are in line with more recent experiments suggesting no relevant contribution of upregulation of classical BAT to cachexia [15].

Only one study investigated the longitudinal behavior of BAT activity on multiple FDG PET scans in a small cohort of 33 breast cancer patients. While confirming the association of BAT activity and BMI, these patients exhibited a rather random BAT activation during the course of neoadjuvant chemotherapy without any correlation to changes of tumor burden [28]. This is in line with our finding that BAT activity is associated with a lower BMI but that increase or decrease in BAT activity does not correlate with active tumor burden. On the contrary, our pilot study had suggested a (ultimately spurious!) negative correlation between an increase in tumor burden and BAT activity in Cohort I. Given the low prevalence of active BAT on FDG PET/CT scans without BAT stimulation of 8–13% [24,41–43] the initial cohort of 1 060 scans was apparently too small to assess a conclusive relation between cancer and BAT activity. We still opted to display the cohorts I and II separately to a) document our process, and b) emphasize the need for large numbers in retrospective studies of this kind. With the substantial increase of the longitudinal dataset from 33 to 283 cancer patients with BAT on serial FDG-PET/CT scans, the initially suggestive negative correlation between changes in metabolically active tumor burden and BAT had vanished.

There are several limitations to our study that need to be acknowledged: Inherent to any retrospective study is a selection bias. In our case, it needs to be noted in particular that in routine clinical practice, certain cancers as well as generally terminally ill and cachectic patients are only rarely re-staged by FDG-PET/CT. This explains the rather low number of severely underweight patients in our cohort. However, using longitudinal data we mitigated this shortcoming by assessing the whole process of weight loss during the course of the disease, independent of BMI threshold or other artificial criteria for cachexia. Although the number of patients with longitudinal data could be increased by extending our inclusion window, the selection bias would persist. Nevertheless, if a positive trend between tumor activity and BAT had been observed in this longitudinal dataset, a prospective trial with a series of additional low dose FDG scans with stimulated BAT (cold exposure and β_3_-agonists) would have been a reasonable next step to shed further insights into the relation between cancer and BAT activity. However, given that we did not find any association between BAT activity and tumor burden, we believe this intervention is not justified.

Next, our analysis was based solely on FDG-PET/CT, which has the inherent limitation of only visualizing and measuring glycolytic activity. Therefore, it is theoretically possible that other brown or beige/brite adipocyte populations, which cannot be evaluated using FDG-PET, might still contribute to the development of cachexia. Moreover, there are numerous other factors associated with BAT activation, such as physical activity, underlying endocrine disorders or administered medications, which introduce possibility of hidden confounders. Given the fact that we found a strong statistical signal between temperature drop and BAT activation, we hypothesize that if present, the large number of subjects should have nonetheless allowed us to detect a signal between cancer evolution, BAT activation and weight loss.

In summary, after several mouse models had shown that the pathological activation of BAT was associated with cancer cachexia [33–40], the retrospective results from cross-sectional human studies [28–31], also suggested an association between cancer and BAT activity. Hence, the general notion that cancer-induced weight loss may partly be due to pathological BAT activation gained increasing acceptance [14]. Our large retrospective longitudinal cohort study did not confirm this hypothesis.
